# Computed tomography angiography of the coronary arteries: major findings in the clinical routine of a general hospital

**DOI:** 10.1590/0100-3984.2020.0047

**Published:** 2021

**Authors:** Rafael Mansur Souto, Alair Augusto Sarmet Moreira Damas dos Santos, Marcelo Souto Nacif

**Affiliations:** 1 Hospital Universitário Antônio Pedro - Universidade Federal Fluminense (HUAP-UFF), Niterói, RJ, Brazil.

**Keywords:** Multidetector computed tomography, Coronary angiography/methods, Coronary artery disease/diagnostic imaging, Tomografia computadorizada multidetectores, Angiografia coronária/métodos, Doença da artéria coronariana/diagnóstico por imagem

## Abstract

Almost two decades ago, it became possible to use coronary computed tomography for the noninvasive assessment of the coronary arteries. That is an extremely accurate method for detecting or excluding coronary artery disease, even the subclinical forms. This pictorial essay aims to show the main imaging findings in 47 coronary computed tomography scans acquired at a general hospital between January 2014 and June 2018. The most common findings were atheromatous plaques (in 87%) and stents (in 34%). There were also incidental findings, not directly related to coronary artery disease, such as pulmonary nodules and aortic stenosis.

## Abstract (pt)

Há quase duas décadas, tornou-se possível a avaliação não invasiva das coronárias por meio da angiotomografia. Esta é bastante importante para excluir ou detectar doença arterial coronariana, mesmo que subclínica. Este ensaio tem o objetivo de mostrar os principais achados de imagem em 47 angiotomografias realizadas de janeiro de 2014 a junho de 2018 em um hospital geral. Os achados mais frequentes foram a presença de placas ateromatosas (87%) e *stents* (34%). Além desses, houve também achados incidentais não relacionados diretamente com doença arterial coronariana, como nódulos pulmonares e estenose aórtica.

## INTRODUCTION

Nearly two decades ago, it became possible to use computed tomography angiography (CTA) for noninvasive assessment of the coronary arteries, as an extremely accurate method for detecting or excluding coronary artery disease (CAD), even the subclinical forms^(1,2)^. Studies have shown that CTA has a high negative predictive value (96-100%), meaning that it is a reliable method of excluding stenosis of the coronary arteries, as well as of CAD^(3)^.

This pictorial essay aims to show the main imaging findings on coronary computed tomography. To that end, we evaluated coronary computed tomography scans acquired at a general hospital between January 2014 and June 2018.

## IMAGING FINDINGS

We evaluated the coronary computed tomography scans of 47 patients. The main findings were atheromatous plaques, in 41 patients (87%); stents, in 16 (34%); myocardial bridging in the anterior descending artery (ADA), in three (6.3%); myocardial fibrosis, in three (6.3%); and obstruction of a coronary segment by a thrombus, in one (2.1%). Among the secondary findings reported were an anomaly of origin and course, in one patient (2.1%); aortic stenosis, in two (4.2%), and pulmonary nodules, in one (2.1%).

Although intracoronary ultrasound is the main imaging method for the evaluation of coronary plaque components, there have been studies showing that CTA can be quite useful in defining plaque components by means of specific quantification in Hounsfield units^(4)^. On CTA, calcified and noncalcified plaques can be evaluated, as can signs of vulnerability, such as lipid content in the plaques, as well as stents and revascularization. In 2016, the Coronary Artery Disease-Reporting and Data System (CAD-RADS) was established to standardize reports and to improve communication between radiologists and requesting physicians^(5)^. The CAD-RADS divides involvement on radiology reports into five grades, ranging from CAD-RADS 0 (the absence of plaques and luminal narrowing) to CAD-RADS 5 (total obstruction of coronary segments).

**Figure 1:**
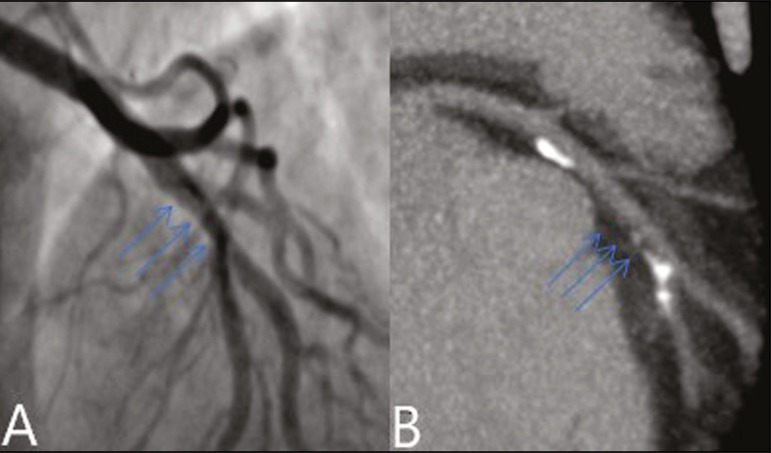
Mixed plaque, resulting in discrete stenosis in the ADA (CAD-RADS 2). **A:** Analysis by catheterization. **B:** Analysis by CTA.

**Figure 2:**
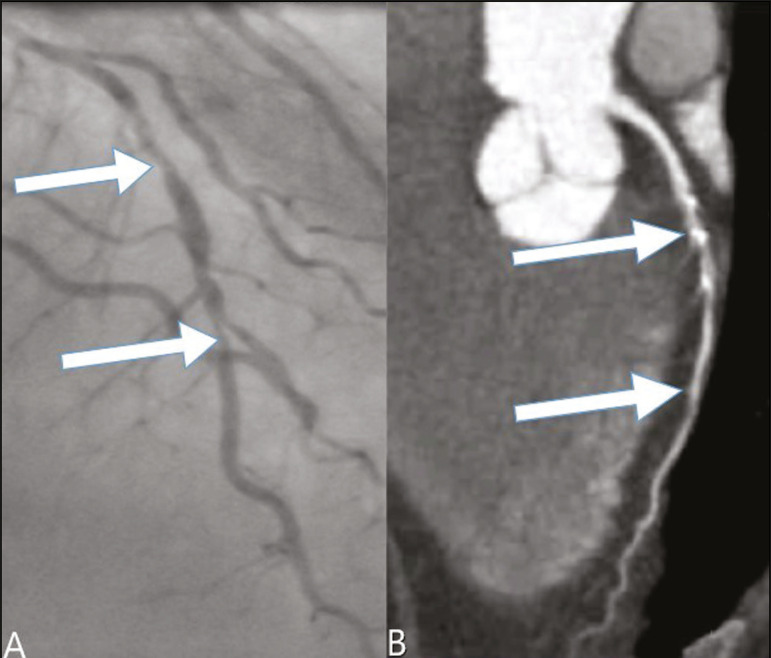
Mixed plaque, resulting in 70% stenosis in the proximal segment of the ADA, and noncalcified plaque, resulting in 90% stenosis in the distal segment of the ADA (CAD-RADS 4A). **A:** Analysis by catheterization. **B:** Analysis by CTA.

**Figure 3:**
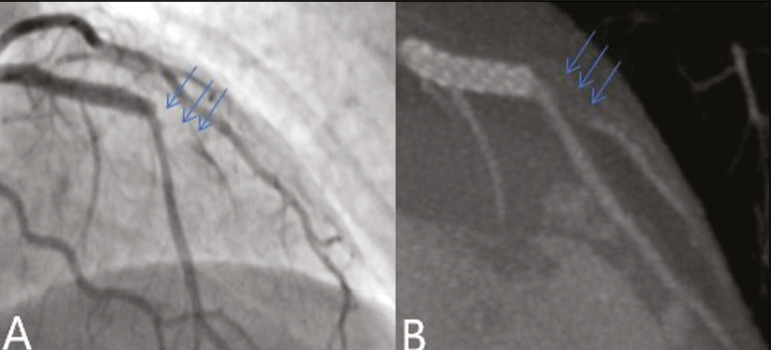
Patient with an unobstructed stent in the ADA, together with thrombus and occlusion at the origin of the diagonal branch of the ADA. In this case, the modifier S (for “stent”) is added (CAD-RADS 5S). **A:** Catheterization. **B:** CTA.

**Figure 4:**
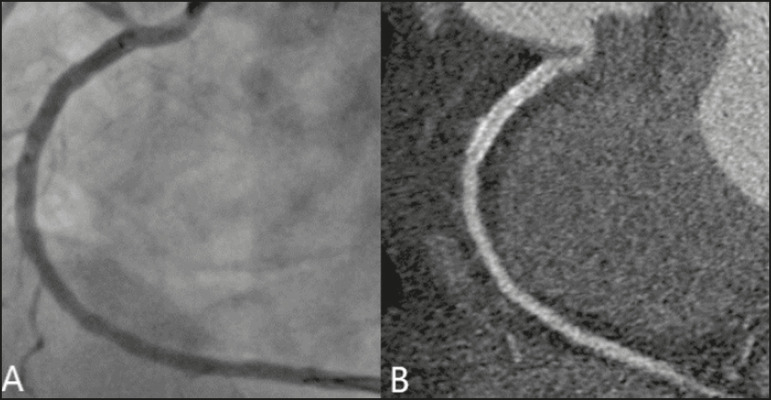
Unobstructed stent in the right coronary artery. **A:** Catheterization of the right coronary artery. **B:** CTA of the right coronary artery.

In our study, the radiology reports were standardized according to the CAD-RADS (Figures 1 and 2). On some of the CTA scans included in this study, it was also possible to evaluate stents and thrombi (Figures 3 and 4), which is another important function of the method.

**Figure 5:**
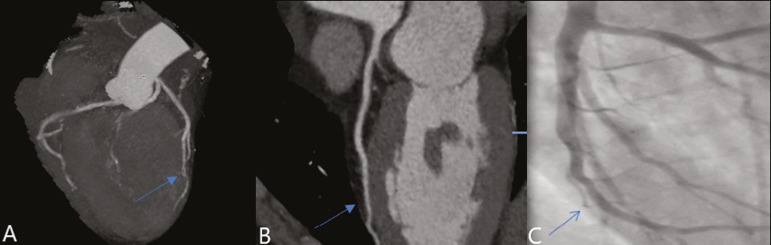
Myocardial bridge in the ADA. **A:** Maximum intensity projection reconstruction. **B:** CTA. C: Catheterization.

As previously noted, myocardial bridging in the ADA was the third most common finding in our sample. It is known that myocardial bridging is a congenital anomaly in which the myocardium crosses over or envelops a segment of an epicardial artery and may manifest as angina pectoris^(6)^, which is one of the main differential diagnoses of CAD. The area below a myocardial bridge is spared from atherosclerotic disease, whereas the proximal area presents a risk of CAD development, which is extremely important in the clinical context of this study. In this study, we included an example of a myocardial bridge in the ADA without accompanying signs of CAD (Figure 5).

**Figure 6:**
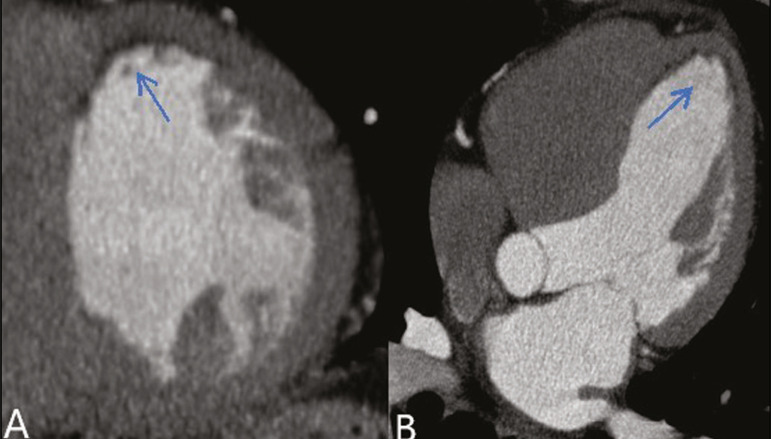
**A,B:** Fibrosis and fatty degeneration in the anterior myocardial wall and apical region of patients with significant CAD. Blue arrow: characteristic hypoattenuation.

Myocardial fibrosis and fatty degeneration can also be the result of acute myocardial infarction and are often seen on cardiac magnetic resonance imaging. Those findings can be seen on CTA, because the kinetics of iodinated contrast in infarcted and non-infarcted areas follow patterns similar to those of gadolinium. The evaluation of myocardial fibrosis is of extreme importance for the determination of myocardial viability; areas of fibrosis can be seen on CTA, as well as on images acquired with functional methods such as cardiac magnetic resonance imaging^(7)^. Figure 6 exemplifies one such case.

**Figure 7:**
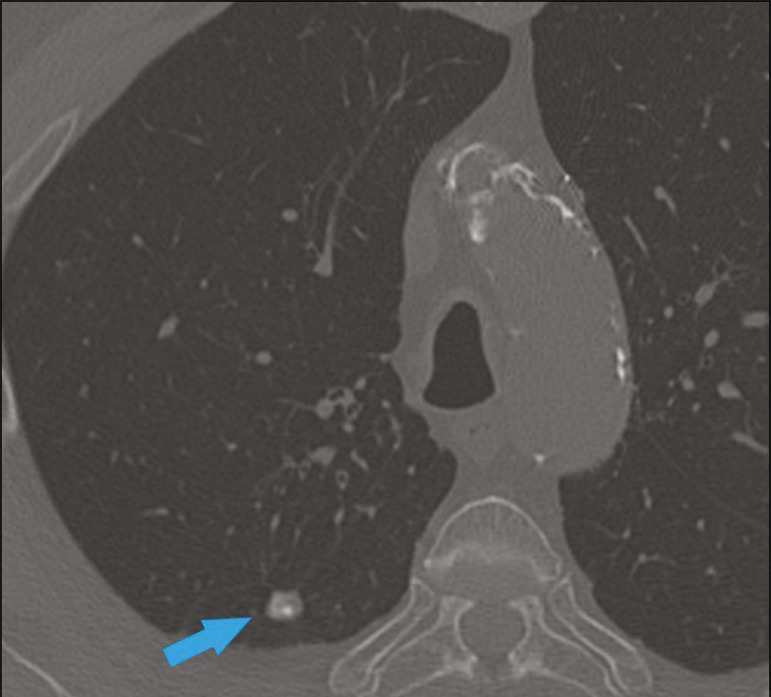
Single coronary artery involving the origins of the ADA and right coronary artery (RCA). **A:** Single coronary artery (CA) originating from the right sinus of Valsalva; origin of the ADA (arrow) showing its course anterior to the aorta. **B:** RCA and ADA. **C:** Single CA originating from the right sinus of Valsalva, giving rise to the RCA and ADA. The ADA presents an intramyocardial course anterior to the aorta.

Although anomalous coronary arteries are reported to be present in only 0.3-5.6% of the population and are less common than are acquired CADs, they have been associated with sudden death in young individuals^(8)^. There are anomalies in which there is a single coronary artery on the left that courses anterior or posterior to the aorta. The CTA of one of the patients identified an anomalous coronary artery in which there was a single coronary artery encompassing the left and right coronary arteries. The ADA presented an intramyocardial course anterior to the aorta (Figure 7).

**Figure 8:**
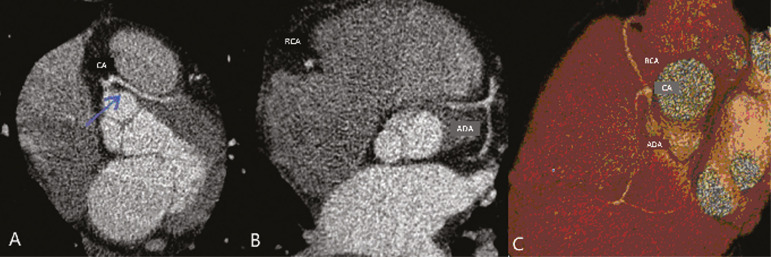
Right pulmonary nodule (blue arrow) on CTA.

**Figure 9:**
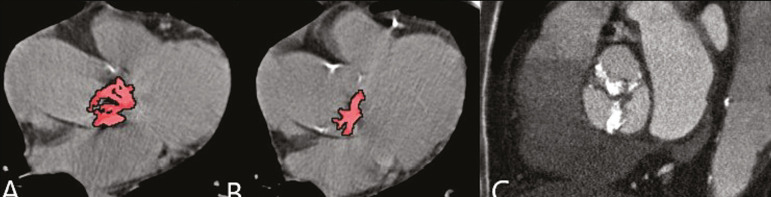
Calcifications in the aortic valve. **A,B:** The red color demonstrates the tool for calculating the calcium score (Agatston score). Reported Agatston score: 8,076. **C:** Aortic valve with calcifications.

When the calcium score is estimated from the images, CTA reveals incidental findings, such as pulmonary nodules (Figure 8), in 0.6-2.4% of cases^(9)^. The frequency of such findings in our study was 2.1%. In addition, 4.7% of the incidental findings on CTA are caused by vascular disorders^(9)^. Aortic stenosis, characterized by multiple calcifications in aortic valve leaflets, may be one of the incidental findings in the coronary evaluation, as in the case exemplified in Figure 9. In that case, the calcium score (Agatston score) for the aortic valve was 8,076. According to the current guidelines, an Agatston score above 1,650 is one of the predictors of significant aortic stenosis^(10)^.

## CONCLUSION

In summary, most of the radiological findings illustrated in this pictorial essay, which were seen in the clinical routine of a general hospital, were directly or indirectly related to CAD, including coronary arterial plaques, thrombi, and stents, as well as myocardial fibrosis. However, there are certain incidental findings, such as pulmonary nodules, which, despite being less common, may improve clinical outcomes, because their discovery can avoid diagnostic delays.
